# Controlling the motion of gravitational spinners and waves in chiral waveguides

**DOI:** 10.1038/s41598-023-50052-0

**Published:** 2024-01-12

**Authors:** A. Kandiah, I. S. Jones, N. V. Movchan, A. B. Movchan

**Affiliations:** Department of Mathematical Sciences, University of Liverpool, Liverpool, L69 7ZL UK

**Keywords:** Applied mathematics, Mechanical engineering

## Abstract

In this paper we present a mathematical modelling framework for chiral phenomena associated with rotational motions, highlighting the combination of gyroscopic action with gravity. We discuss new ideas of controlling gravity-induced waves by a cluster of gyroscopic spinners. For an elementary gravitational spinner, the transient oscillations are accompanied by a full classification and examples, linked to natural phenomena observed in planetary motion. Applications are presented in the theory of chiral metamaterials, and of the dynamic response of such materials to external loads.

## Introduction

The theme of *Chirality in Nanomaterials* covers a range of physical phenomena at different scales: atomic scale (elementary particles), molecular scale (chemistry), macroscale (mechanical lattices in physics and engineering) as well as megascale (planetary systems and galaxies). Spiral galaxies are among the most common types of galaxies in the universe, and they exhibit a distinct handedness (chirality) in their shape and rotation. We note the diversity and complexity of chirality phenomena across different scales and disciplines. A formal link between models of galaxies and microscopic systems, such as aminoacids, sugars, neutrinos, was discussed by Capozziello and Lattanzi^1^.

Often, chiral systems are considered as geometrical objects which are non-superimposable on their mirror image. Examples of such systems are widely available in molecular structures. On the other hand, the fundamental laws of physics include examples of dynamic chirality (also referred to as physical chirality) associated with the spiral motion of charged particles in an ambient magnetic field. Such a spiral motion is induced by the Lorentz force (see^2^), which is orthogonal to the velocity vector of the moving particle. In mechanics, the gyroscopic force produces an effect similar to that of the Lorentz force in problems of electromagnetism. The direction of the gyroscopic motion is linked to the orientation of the mechanical spinner. An important example of a multi-scale rotational gyroscopic system is the Solar System, which also incorporates the force of gravity.

The phenomenon of physical chirality is investigated in the present paper. The emphasis is on the dynamic response of a multi-body gyroscopic system, which can be considered at different scales and for different types of motion. This is in contrast with geometrical chirality of static objects. The force of gravity has a pronounced effect on the motion of a spinner or a cluster of connected spinners, and this is in the main focus of the paper.

The notion of gravitational spinners takes into consideration the combined action of gyroscopic forces and gravity. In mechanics, the transient processes for such systems are of particular interest, due to formal connections with a range of natural phenomena, linked to planetary rotational motions. Well-known examples include the Foucault pendulum^3^, polygonal patterns of Rossby waves^4^, hexagonal shape of vortex flows at the North Pole of Saturn^5^ and polygonal patterns of cyclones in polar observations of Jupiter^6^. Additionally, the notion of chirality in physics and mechanics has been established in the classical literature, such as the books by Lord Kelvin^7^, Webster^8^ and Gray^9^. The recent monograph by Kirillov^10^ has addressed chiral motion in the context of non-conservative stability problems.

In 1851, Foucault proposed a mechanical system, incorporating a pendulum of sixty-seven metres in length^3^, which could be used to demonstrate rotation of Earth. The Foucault pendulum shows precession, similar to that observed in a gyroscope. Although the precession of the Foucault pendulum is very slow, with the overall period being $(24~ \text{ h})/\sin (\phi )$, where $\phi$ is the latitude relative to the equator, the linearised governing equations are similar to those describing the pendulum with the gyroscope attached at its end (as in^9^). In the latter case, the motion can be controlled by changing the rate of spin of the gyroscope, the moments of inertia and the initial conditions. As discussed by Nash et al.^11^, for a cluster of gyroscopic pendulums, the transient behaviour of the mechanical system is more complex and may show preferential directions of the edge wave along the boundary of the cluster.

The theoretical background for modelling the dynamic response of elastic multi-structures, combined with the gyroscopic spinners, is included in Carta et al.^12–15^, Nieves et al.^16^, and Kandiah et al.^17^. In particular, the articles^12–16^ focus on the analysis of effective boundary conditions, which produce the gyroscopic action, for elastic systems such as flexural beams, in the cases of time-harmonic and transient motion. These papers have no gravity terms in the governing equations, and the main results combine the elastic response of the overall structure and the gyroscopic moment incorporated into the boundary conditions. The article^17^ takes into account gravity in combination with the angular momentum for the chiral systems.

In the present paper, we discuss the classification of chiral waveguides, periodic and non-periodic, as well as the methods of controlling dynamics of gravitational spinners: firstly, by analysing the wave dispersion and Green’s kernels for a periodic cluster of gravitational spinners, and secondly, by classifying all possible trajectories of individual gravitational spinners using the angular momentum method. Experimental measurements are compared with the analytical approximations. In addition to the analytical work and numerical illustrations, the paper also includes electronic supplementary materials with animated motions (Supplementary Videos V1, V2, V3).

## Chiral waveguides: classification and control of the wave dispersion

The discrete models of elastic chiral metamaterials are presented in^14,18^. Gyroscopic spinners are embedded into a doubly periodic triangular elastic lattice, in the absence of gravity. In statics, no chirality whatsoever is observed in such a system. However, the gyroscopic spinners embedded into the triangular lattice deliver strong dynamic anisotropy for Floquet-Bloch waves, and their dispersion is affected significantly by the dynamic chirality of the system. A special type of standing wave, called a *vortex wave*, emerges in the two-dimensional elastic lattice with identical gyroscopic spinners. These waves are characterized by a circular motion of the lattice nodes, which matches the rotation of the gyroscopic spinners.

The effect of gravity is often neglected in many mathematical models of waves in elastic systems. We show the importance of the combined contribution from gravity and gyroscopic chirality for the propagation of waves. Here, we focus on the uniaxial waveguides, where the waves travel along one direction, but with a “twist”: these waveguides are gyroscopic, and there is a chiral coupling between the velocity components.

### Continuous chiral waveguides subjected to gravity

Two types of continuous chiral gyroscopic waveguides are outlined here: horizontal uniaxial waveguides orthogonal to the direction of the force of gravity, and vertical uniaxial waveguides aligned with the direction of the force of gravity. In both cases, the rotational motion occurs due to the presence of gyroscopic spinners, but gravity makes a significant difference between these two cases.

#### Uniaxial gyroscopic elastic waveguides orthogonal to the direction of the force of gravity

The governing equations are obtained as a result of the homogenisation procedure for a periodic system of vertically suspended gyroscopic pendulums, connected by horizontal massless pre-stressed elastic springs, along the *x*-axis. Assuming that *u*(*x*, *t*) and $v(x,t)$ are the longitudinal and transverse displacement components in the horizontal plane, in the linear approximation the governing equations have the form

1 $$\frac{\partial ^2 u}{\partial t^2}-\frac{c_1}{\rho }\frac{\partial ^2 u}{\partial x^2} -\alpha \frac{\partial v}{\partial t}+Gu = 0 , ~~ \frac{\partial ^2 v}{\partial t^2}-\frac{c_2}{\rho } \frac{\partial ^2 v}{\partial x^2} +\alpha \frac{\partial u}{\partial t}+Gv = 0, $$

where $c_1, c_2$ are normalised stiffness coefficients, $\alpha$ is the gyroscopic chirality parameter, $\rho$ is the mass density, and *G* is the normalised gravity parameter (for the case of a pendulum, $G= g/L$, where *g* is the gravity acceleration, and *L* is the arm length of the pendulum); the variable *t* represents time. We note that when $\alpha =G=0$, i.e. no gyroscopic chirality and gravity are present, Eq. (1) becomes a system of decoupled wave equations. The terms representing the gyroscopic action, couple the components of the velocity in the horizontal plane, due to the gyroscopic force being orthogonal to the velocity vector.

It is also noted that when the parameter $\alpha$ is increasing, over a finite time interval the solution becomes confined in the spatial dimension, and rapid temporal variations are observed. Here, gravity appears to be an important control parameter, which enables one to control the oscillatory character of the coupled displacement components.

**Figure 1:**
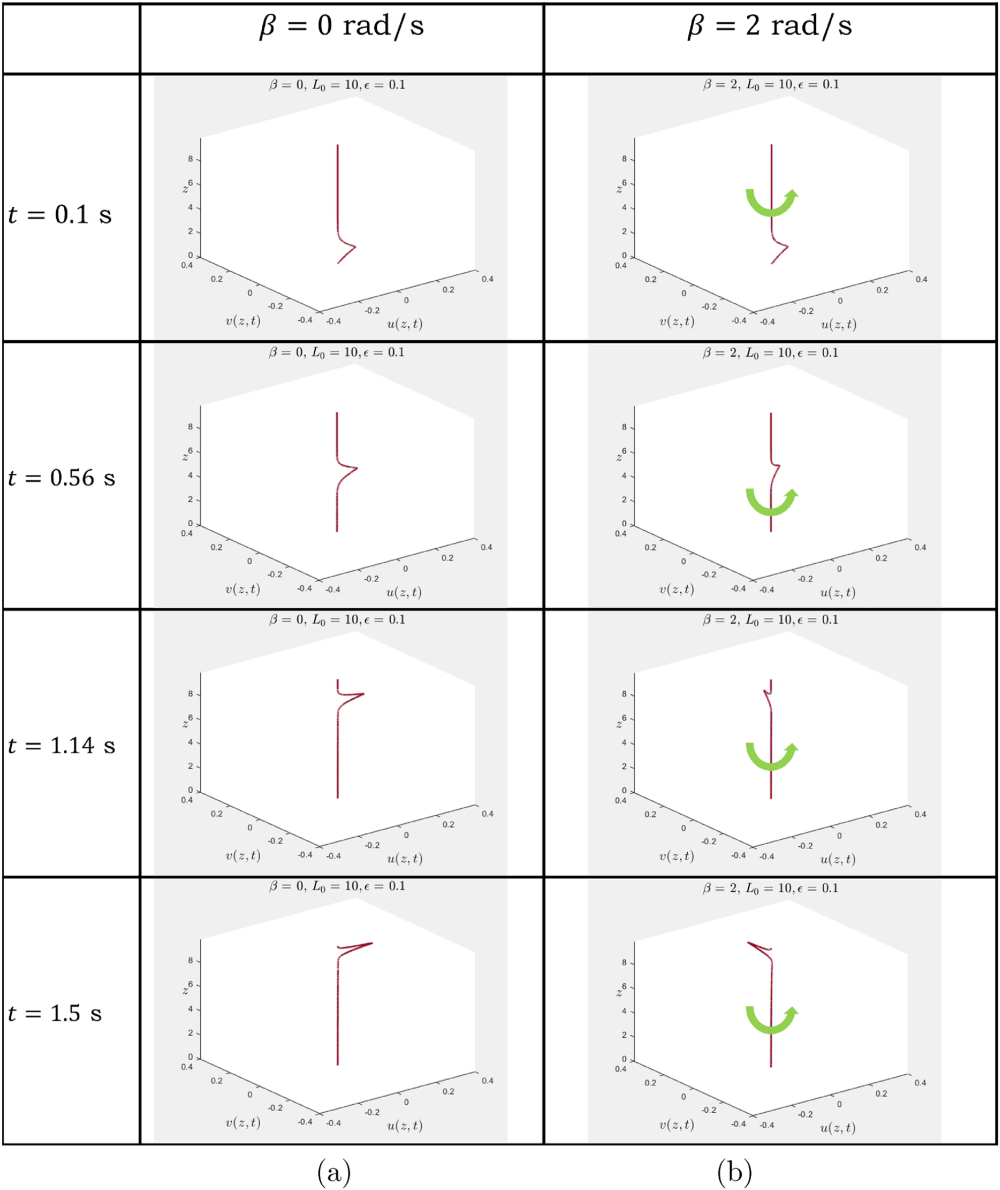
Gravity-induced waves in a gyroscopic waveguide: (**a**) $\beta =0$, no chirality; (**b**) $\beta =2$, rotational motion is observed along the waveguide—the arrow shows the orientation of the rope rotation.

#### Non-elastic gyroscopic waveguides aligned with gravity

The problem becomes very different when the waveguide is positioned vertically, being aligned with the direction of the force of gravity. If one thinks of a vertical chain of gyroscopic pendulums along the *z*-axis, with the positive direction of the *z*-axis aligned with the direction of the force of gravity, in the homogenisation limit the governing equations for the transverse displacements *u*(*z*, *t*) and $v(z,t)$ become

2 $$\begin{aligned} \frac{\partial ^2 u}{\partial t^2} - g(L_0 - z)\frac{\partial ^2 u}{\partial z^2}+\beta \frac{\partial v}{\partial t}+g\frac{\partial u}{\partial z} = 0, ~~ \frac{\partial ^2 v}{\partial t^2} - g(L_0-z)\frac{\partial ^2 v}{\partial z^2}-\beta \frac{\partial u}{\partial t}+ g \frac{\partial v}{\partial z} = 0, \end{aligned}$$

where *u*(*z*, *t*) and $v(z,t)$ are the transverse displacement components of a *gyroscopic vertical rope of length* $L_0$, and *g* is the gravity acceleration. In Eqs. (2), the quantity $\beta$ is referred to as the chirality control parameter, which can be positive or negative, depending on the orientation of the gyroscopic spin.

Equation (2) describe waves, which are induced by gravity: no elastic resistance is present in the waveguide. Compared to (1), the differential equations governing the motion of a gyroscopic, vertically hanging rope, include variable coefficients, which depend on the spatial variable along the rope. The gyroscopic coupling between the transverse displacement components induces rotational motions of points along the rope, as the wave propagates, with a variable speed, along the waveguide. Also, the gravity-dependent terms in (2) are proportional to the spatial derivatives of the transverse displacements *u* and $v$, in contrast with (1) where the gravity is represented by the terms *Gu* and $Gv$.

Equation (2) are related to the classical problem of a vertically suspended rope, subjected to gravity, which in the absence of gyroscopic chirality is well-known and its study was initiated by the work of Bernoulli and Euler (see the historical account in^19^). The new chiral terms bring an additional feature of rotational motion, illustrated in Fig. 1. Also, solutions of (2) may exhibit a logarithmic singularity at $z=L_0.$ The Dirichlet boundary conditions can be prescribed at $z=0$ and $z=L_0-\epsilon , ~ 0< \epsilon \ll 1,$ and such a problem is singularly perturbed in the limit as $\epsilon \rightarrow 0$.

An illustrative example of a transient motion of the gravity-induced wave is shown in Fig. 1. The end of the rope at the pivot point $z=0$ is fixed, and the rope is also fixed at $z=L_0 - \epsilon$, where $\epsilon$ is a small positive parameter. The model represents a singular perturbation problem, with the boundary layer observed near the end of the rope. At the initial time $t=0$, an exponentially localised velocity along the *x*-axis is set to trigger the wave. Accordingly, the boundary conditions, used in the numerical simulation, are $u(0, t) = v(0, t) = 0,$ and $u(L_0-\epsilon , t) = v(L_0-\epsilon , t) = 0,$ where $\epsilon = 0.1$ and $L_0=10$. The initial conditions are $u(z, 0) = v(z,0) = 0$ and $\frac{\partial u}{\partial t} (z, 0) = 2 \exp (-z^2), \frac{\partial v}{\partial t} (z, 0) = 0.$

In Fig. 1a, the chirality control parameter is set to be zero (no gyroscopic coupling), and the wave of the same polarisation, characterised by the deflection in the (*x*, *z*)-plane moves along the *z*-axis. In part (b) of the same figure, where $\beta =2$, the gyroscopic chirality leads to the rotation of the wave as it propagates along the *z*-axis. The videos, which include the animation of the gravity-induced waves, are in the electronic supplementary material available with this paper.

### Chiral structured waveguides: gravity control of the wave dispersion

It is known that continuous and discrete (also referred to as “structured”) waveguides show different dynamic responses in the context of the wave dispersion.

**Figure 2:**
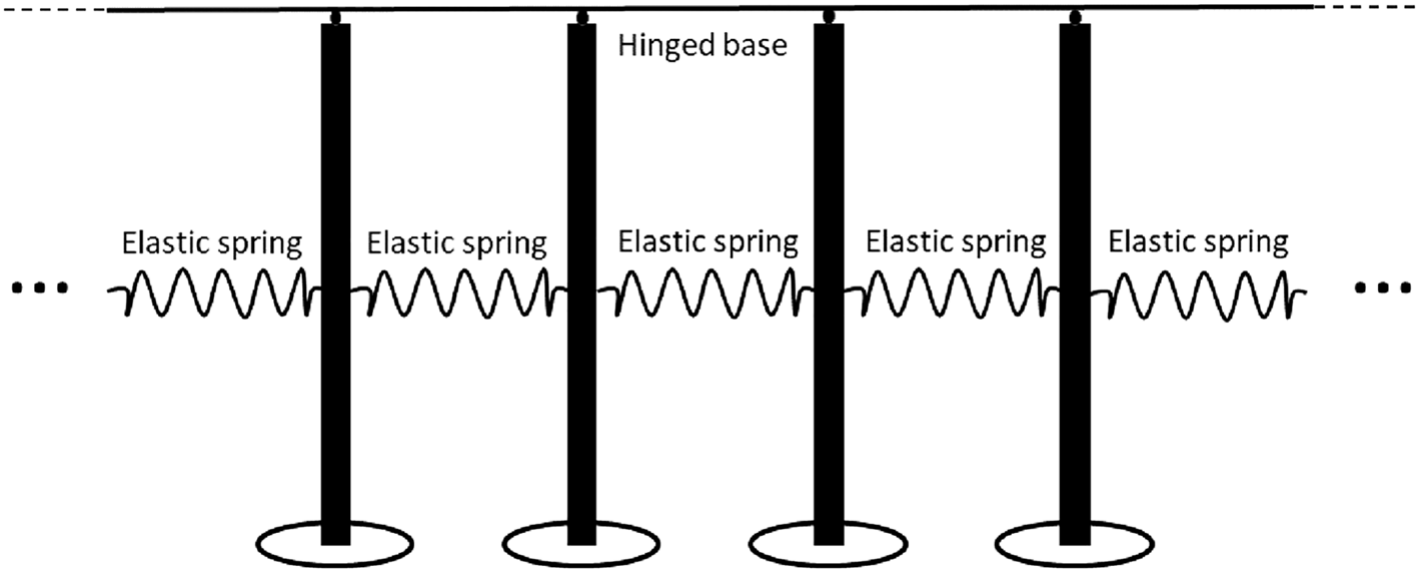
Gyroscopic structured chiral waveguide.

Here, instead of the continuous system, described by (1), we consider a periodic chain of gravitational spinners, as shown in Fig. 2, which forms a chiral structured waveguide. Such a structure is relevant to the theory of chiral metamaterials, subjected to the control of the wave localisation and wave dispersion.

The connections between the spinners are maintained by massless springs, and the gyropendulums are positioned at $x=n$, $n\in \mathbb {Z}.$ The Fourier transform in time is formally applied, and here we assume that the chiral system is active, similar to that in^17,18,20^, with the chirality parameter $\alpha$, the vector amplitude ${\textbf {U}}^{(n)} =(U_1^{(n)},U_2^{(n)})^{T}$ of the time-harmonic motion at the nodal point $x=n$, and the radian frequency $\omega$. The system of governing equations, written in the vector form, becomes

3 $$\begin{aligned} -m\omega ^2 {\textbf {U}}^{(n)}={\textbf {C}}({\textbf {U}}^{(n-1)}+{\textbf {U}}^{(n+1)}-2{\textbf {U}}^{(n)})+i\alpha m\omega ^2{\textbf {R}}{} {\textbf {U}}^{(n)}-mG{\textbf {U}}^{(n)}, \end{aligned}$$

where *G* is the normalised gravity parameter, the rotation matrix ${\textbf {R}}$ is given by

4 $$\begin{aligned} {\textbf {R}}=\begin{pmatrix} 0 &{} 1\\ -1 &{} 0 \end{pmatrix}, \end{aligned}$$

and the stiffness matrix is ${\textbf {C}}= \text{ diag }\{ c_1, c_2 \}.$ The quantity $c_1>0$ denotes the elastic stiffness of the springs and similar to^20^, we also allow for a pre-tension in the springs, which is represented by an effective transverse stiffness $c_{2} \ge 0.$

Assuming that the distance between neighbouring nodal points is *a*, and *k* is the wave number, the Floquet-Bloch condition is ${\textbf {U}}^{(n+1)}=e^{ika}{} {\textbf {U}}^{(n)},$ and the system (3) is reduced to the form

5 $$\begin{aligned} \left[ m(G-\omega ^2){\textbf {I}}-2(\cos (ka)-1){\textbf {C}}-i\alpha \omega ^2 {\textbf {R}}\right] {\textbf {U}}^{(n)}={\textbf {0}}. \end{aligned}$$

It is also convenient to use non-dimensional variables, defined by

6 $$\begin{aligned} \tilde{{\textbf {U}}}^{(n)}=\frac{1}{a}{} {\textbf {U}}^{(n)}, \quad \tilde{k}=ka, \quad \tilde{\omega }=\omega \sqrt{\frac{m}{c_1}}, \quad \tilde{c}=\frac{c_2}{c_1}, \quad \tilde{G}=\frac{mG}{c_1}. \end{aligned}$$

Dropping tilde (for the sake of convenience) in the following text, the dispersion equation can be written in the form

$$\begin{aligned} (1-\alpha ^2)\omega ^4-2((c+1)(1-\cos k)+G)\omega ^2+G^2 \end{aligned}$$

7 $$\begin{aligned} +2(1-\cos k)(c+1)G+4c(1-\cos k)^2 =0. \end{aligned}$$

Here, it is assumed that $0 \le \alpha < 1$. This gives two dispersion curves defined by

8 $$\begin{aligned} \omega ^{\pm }=\Big (Q^{\pm }(\alpha ,c,k,G)\Big )^{1/2}, \end{aligned}$$

where

$$\begin{aligned} Q^{\pm }(\alpha ,c,k,G)= \frac{1}{(1-\alpha ^2)}\Big (G+(1-\cos k)(c+1) \pm \Big \{(G+(1-\cos k)(c+1))^2 \nonumber \\ -(1-\alpha ^2)(G^2+2G(1-\cos k)(c+1)+4c(1-\cos k)^2)\Big \}^{1/2} \Big ). \end{aligned}$$

### Gravity and gyricity: control of dispersion

For the chiral waveguide, gravity and gyricity, acting together, control the dispersion of the Floquet-Bloch waves, which also includes wave localisation and formation of standing vortex waves.

**Figure 3:**
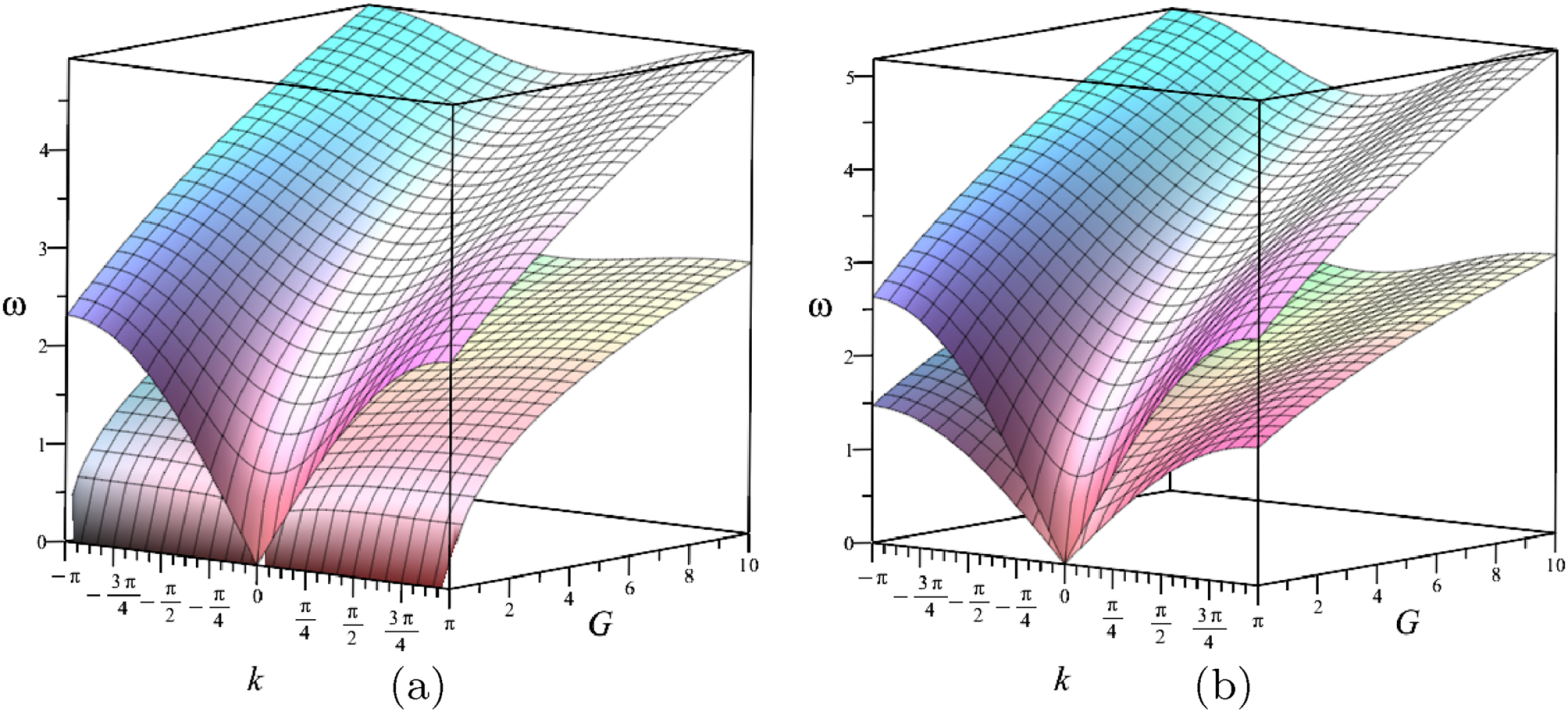
The graphs of $\omega ^{\pm }$ as functions of *k* and *G* for $\alpha =0.5$; (**a**) $c=0$ and (**b**) $c=0.7$.

For the waveguide, subjected to gravity, with pre-tension but without spinners ($\alpha =0$), the equations describing the dispersion curves reduce to an elementary form

9 $$\begin{aligned} \omega ^{+}=\sqrt{G+2(1-\cos k)}, \quad \omega ^{-}=\sqrt{G+2c(1-\cos k)}, \end{aligned}$$

which also show that when $G>0$, there is a finite width band gap adjacent to $\omega =0$. When $\alpha =0$ and $k=0$, it follows that $\omega ^{+}=\omega ^{-}= \sqrt{G}$. Introducing the non-zero chirality parameter $0<\alpha <1$, and using (7), (8), we observe that $\omega ^{+}>\omega ^{-}>0$ for any *k*, provided that $0\le c<1$ and $G>0$. The surfaces $\omega = \omega ^\pm (k, G)$ for $\alpha =0.5$ and two values of *c* are shown in Fig. 3; the change in the dispersion curves can be seen as variations of cross-sections when *G* is fixed.

A special feature of the chiral waveguide, formed by gravitational spinners, is in the presence of several frequency regimes, which we refer to as “total pass band”, “partial pass band”, and “stop band”.

The term *total pass band* corresponds to an interval of values of the radian frequency $\omega$ where both types of Floquet-Bloch waves corresponding to the branches (8) occur, i.e. $\omega ^{+}|_{k=0}\le \omega \le \omega ^{-}|_{k=\pi }$. The frequency interval is referred to as *partial pass band* when only one type of the Floquet-Bloch wave (corresponding to one of the branches $\omega ^{\pm }$) can propagate. For intervals of frequencies, where there are no propagating waves, and all waveforms are evanescent, the term *stop band* is used.

**Figure 4:**
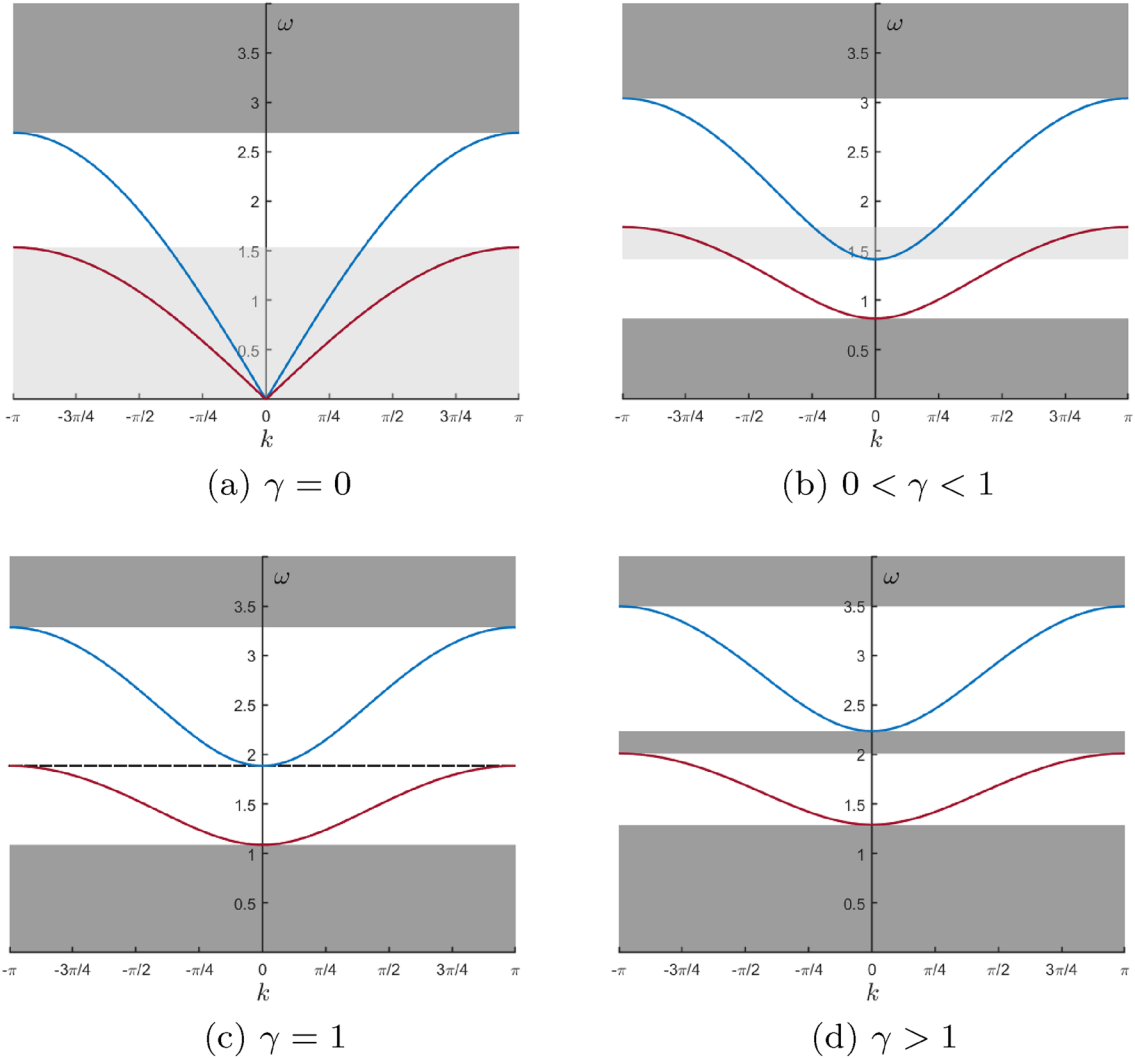
Dispersion diagrams for (**a**) $\gamma =0$, (**b**) $\gamma =0.563$, (**c**) $\gamma =1$ and (**d**) $\gamma =1.406$. In the calculations the following parameters are chosen: $c=0.8$, $\alpha =0.5$ and $G=0, 1, 1.778$ and 2.5 [in parts (**a**–**d**), respectively]. Note that $\gamma = G\alpha (c+1)/(4c(1-\alpha ))$.

In Fig. 4, we show examples of dispersion diagrams where $c=0.8$ and $\alpha =0.5$. It is convenient to introduce the parameter $\gamma =G\alpha (c+1)/(4 c(1-\alpha )),$ and observe the following two cases:

- The case of $0\le \gamma \le 1$ corresponds to $0\le \omega ^{+}|_{k=0}\le \omega ^{-}|_{k=\pi }$ (see Fig. 4a–c); when $\gamma =0,$ we observe one stop band, one total pass band and one partial pass band, when $0<\gamma <1,$ we have two stop bands, one total pass band and two partial pass bands, and when $\gamma =1$ the dispersion diagram is shown in Fig. 4c.
- The case of $\gamma >1$ corresponds to $\omega ^{+}|_{k=0}>\omega ^{-}|_{k=\pi }>0$; in this case there are three stop bands and two partial pass bands (see Fig. 4d).

### Green’s matrix for a gravitational spinner waveguide

Assuming that a time-harmonic force with components $f_{j} e^{-i\omega t}, ~ j=1,2,$ is applied at the origin, where $n=0,$ the inhomogeneous governing equations are given by

10 $$\begin{aligned} \begin{aligned} (G-\Omega ){U}^{(n)}_1=\left( U^{(n+1)}_1+U^{(n-1)}_1-2U^{(n)}_1\right) +i\alpha {\Omega } U^{(n)}_2+\tilde{f}_{1}\delta _{n0}, \\ (G-\Omega ){U}^{(n)}_2=c\left( U^{(n+1)}_2+U^{(n-1)}_2-2U^{(n)}_2\right) -i\alpha \Omega U^{(n)}_1+\tilde{f}_{2}\delta _{n0}. \end{aligned} \end{aligned}$$

Here $\Omega =\omega ^2$ and $\tilde{f}_{i}=f_{i}/ac_{1}.$ The discrete Fourier Transform of Eq. (10) with respect to the Fourier variable *k* leads to

11 $$\begin{aligned} U_{i}^{(n)}=\sum _{j=1,2}{\mathscr {G}}^{(n)}_{ij}\tilde{f}_{j}, ~ i=1,2, \end{aligned}$$

where ${\mathscr {G}}^{(n)}_{ij}$ are components of the Green’s matrix. According to^17^, it can be represented as

12 $$\begin{aligned} {{{\varvec{\mathscr {G}}}}}^{(n)}=F(n,\Omega ,\alpha ,c,G)\left[ (G-\Omega ){\textbf {I}}+i\alpha \Omega {\textbf {R}})\right] -\mathfrak {F}(n,\Omega ,\alpha ,c,G)\text {diag}\{c,1\}, \end{aligned}$$

where ${\textbf {I}}$ is the $2\times 2$ identity matrix, the rotation matrix ${\textbf {R}}$ is given in (4), and

13 $$\begin{aligned}{} & {} F(n,\Omega ,\alpha ,c,G)=\frac{1}{2\pi }\int _{-\pi }^{\pi }\frac{e^{ikn}}{\sigma (\alpha ,\omega ,c,k,G)}dk, \end{aligned}$$

14 $$\begin{aligned}{} & {} \mathfrak {F}(n,\Omega ,\alpha ,c,G)=\frac{1}{\pi }\int _{-\pi }^{\pi }\frac{(\cos k-1)e^{ikn}}{\sigma (\alpha ,\omega ,c,k,G)}dk \nonumber \\{} & {} =F(n+1,\Omega ,\alpha ,c,G)-2F(n,\Omega ,\alpha ,c,G) +F(n-1,\Omega ,\alpha ,c,G), ~\text{ where } \nonumber \\{} & {} \sigma (\alpha ,\omega ,c,k,G)=(1-\alpha ^2)\omega ^4-2((c+1)(1-\cos k)+G)\omega ^2+G^2+2(1-\cos k)(c+1)G+4c(1-\cos k)^2. \end{aligned}$$

Here, we note that the simplified expression for $F(n,\Omega ,\alpha ,c,G)$ depends on the frequency regime, i.e. stop band, partial pass band or total pass band. It is convenient to introduce the quantities:

15 $$\begin{aligned} b^{\pm }=\Bigg (1-\frac{(c+1)(\Omega -G)\mp \sqrt{\Omega ^2(4\alpha ^2c+c^2-2c+1)+G^2(c-1)^2-2G\Omega (c-1)^2}}{4c}\Bigg )^{-1}. \end{aligned}$$

#### The stop band regime

In the *stop band* regime we have $|b^{\pm }|<1$. The components of Green’s matrix are exponentially localised. The function $F(n,\Omega ,\alpha ,c,G)$ in the stop band may be written as

16 $$\begin{aligned} F(n,\Omega , \alpha ,c,G) = \frac{b^- b^+}{4c(b^{-}-b^{+})} \left[ \frac{b^{-}}{\sqrt{1-(b^{-})^2}} \left( \frac{1-\sqrt{1-(b^{-})^2}}{b^{-}}\right) ^{|n|} \right. \nonumber \\ \left. -\frac{b^{+}}{\sqrt{1-(b^{+})^2}}\left( \frac{1-\sqrt{1-(b^{+})^2}}{b^{+}}\right) ^{|n|} \right] . \end{aligned}$$

#### The partial pass band regime

For this regime, the quantities $b^\pm$ satisfy the constraints $|b^{+}|>1$ and $|b^{-}|<1,$ or $|b^{+}|<1$ and $|b^{-}|>1.$ The additional partial pass band region is a special feature that is introduced by gravity. We first consider the case when $|b^{+}|>1$ and $|b^{-}|<1$. Taking into account the radiation condition at infinity, the function $F(n,\Omega , \alpha , c, G)$ in the partial pass band is given by

17 $$\begin{aligned} F(n,\Omega , \alpha ,c,G) = \frac{b^- b^+}{4c(b^{-}-b^{+})}\left[ \frac{b^{-}}{\sqrt{1-(b^{-})^2}} \left( \frac{1-\sqrt{1-(b^{-})^2}}{b^{-}}\right) ^{|n|} -\frac{ie^{i|n|\theta ^{+}}}{\sin (\theta ^{+})}\right] , \end{aligned}$$

where

18 $$\begin{aligned} \cos \theta ^{\pm }=\frac{1}{b^{\pm }}. \end{aligned}$$

The first term in (17) represents the evanescent solution corresponding to the eigenfrequency $\omega ^-$ and the second term represents the propagating solution corresponding to the eigenfrequency $\omega ^+.$ Similarly, for $|b^{+}|<1$ and $|b^{-}|>1$, we can represent the function $F(n,\Omega ,\alpha ,c,G)$ in the partial pass band as

19 $$\begin{aligned} F(n,\Omega , \alpha ,c,G) = \frac{b^- b^+}{4c(b^{-}-b^{+})}\left[ \frac{ie^{i|n|\theta ^{-}}}{\sin (\theta ^{-})}-\frac{b^{+}}{\sqrt{1-(b^{+})^2}} \left( \frac{1-\sqrt{1-(b^{+})^2}}{b^{+}}\right) ^{|n|}\right] , \end{aligned}$$

where the first term in (19) represents the propagating solution corresponding to the eigenfrequency $\omega ^-$ and the second term represents the evanescent solution corresponding to the eigenfrequency $\omega ^+.$

**Figure 5:**
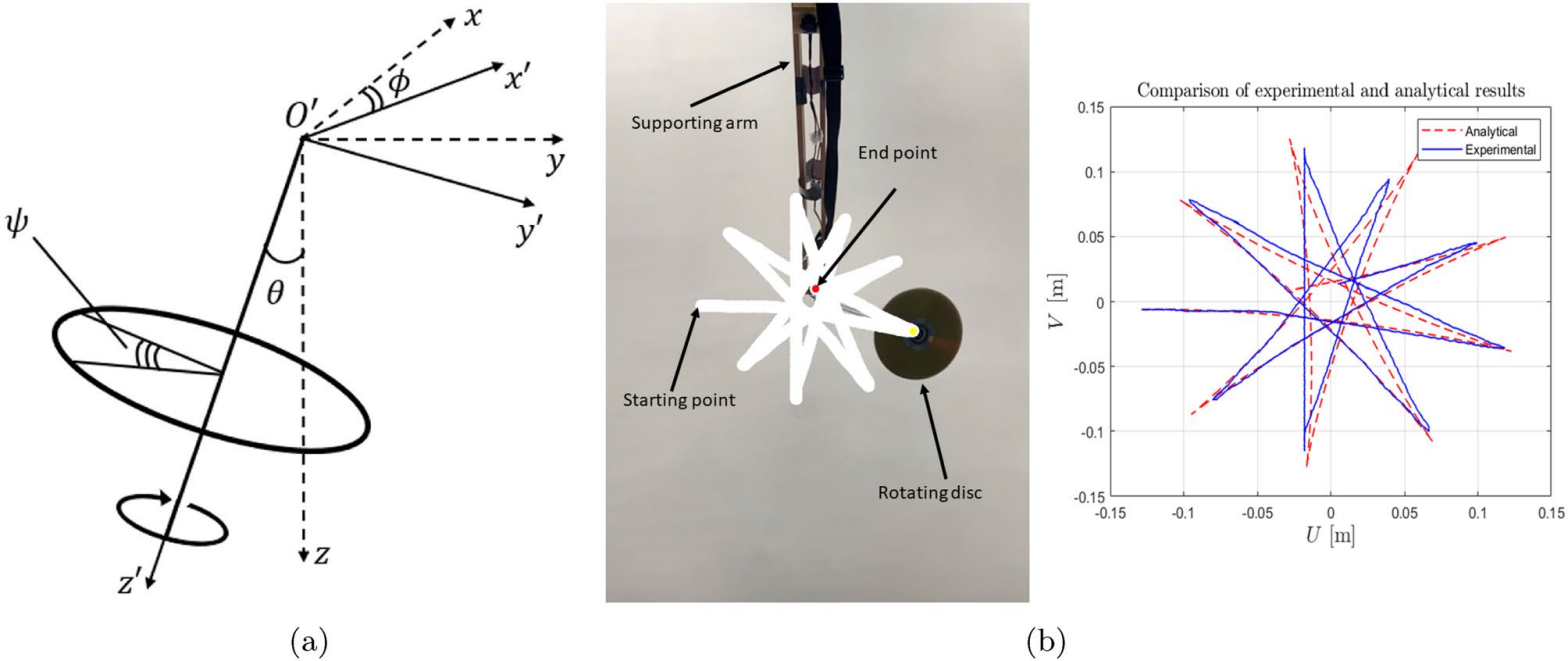
(**a**) A gyropendulum includes a gyroscopic spinner connected to the tip of a rod. The rod is hinged at its base which is located at the origin of the fixed coordinate system *Oxyz*. The gyroscopic spinner is shown in the local coordinate system $O'x'y'z'$, which moves with the spinner as it nutates through an angle $\theta$, precesses through an angle $\phi$ and spins through an angle $\psi$. The axes of the rod and the spinner are assumed to be aligned at any instant of time. (**b**) Experimental observation and analytical prediction for cusp-shaped trajectories; the video of the motion is provided in the electronic supplementary material.

#### The total pass band regime

In the *total pass band* regime, the time-harmonic point force generates two types of outgoing propagating waves. In this region $|b^{\pm }|>1$. The function $F(n,\Omega ,\alpha ,c,G)$ is given in the total pass band by

20 $$\begin{aligned} F(n,\Omega , \alpha ,c,G) = \frac{b^- b^+}{4c(b^{-}-b^{+})} \left[ \frac{ie^{i|n|\theta ^{-}}}{\sin (\theta ^{-})} -\frac{ie^{i|n|\theta ^{+}}}{\sin (\theta ^{+})}\right] . \end{aligned}$$

Both terms in (20) represent propagating solutions that obey the radiation condition at infinity.

## Transient motion of an elementary gravitational spinner

Although gyroscopic multi-structures bring a significant insight into the methods of control of dispersive waves, the transient motion of an elementary gravitational spinner shows counter-intuitive shapes, which can be described analytically. For an elementary single gravitational spinner, a combination of the gyroscopic action and gravity, enable the spinner to “go around the corner”, following trajectories, which approximate polygonal shapes. We demonstrate that other shapes also include cusps, self-intersecting loops and smooth curves, assembled in three classes. In some particular cases, the bounded motions of a gravitational spinner can exhibit trajectories similar to those of the Foucault pendulum, as well as the stable motion of a Brouwer particle moving on a surface rotating with a constant angular velocity as noted in^10,21–24^.

In this section, a model will be described for a gravitational spinner which incorporates both the effect of gravity, as in a pendulum, and a rotational element, as in a gyroscope. The gravitational spinner is shown in Fig. 5a. It consists of a rigid, massless rod of length *L*,  suspended from a pivot at $z=0$, so that it can swing freely under gravity. At $z=L$, the rod is connected to a thin uniform disc of mass *m* and radius *R*,  which acts as a gyroscopic spinner. The spinner is axisymmetric, with centre of mass placed at the end of the rod. There is no external energy flux within the gyroscopic system under gravity and it will be referred to as a passive gyroscopic system. For such a system, the energy is conserved.

The motion of gyroscopic spinners is usually characterised by the angular coordinates $\theta$, $\phi$ and $\psi .$ They are the angles of nutation, precession and spin, respectively. The analysis here is limited to the regime where the nutation angle $\theta$ and its derivatives are considered to be small, resulting in a linearised model. For $0\le z\le L$, the transverse displacements in the *x* and *y* directions are *u*(*z*, *t*) and $v(z,t)$,  respectively, and are linear functions of *z*,

21 $$\begin{aligned} u(z,t)=zU(t), \quad v(z,t)=zV(t), \end{aligned}$$

where *U*(*t*) and *V*(*t*) are time-dependent non-dimensional coefficients associated with the transverse displacement components. In the linearised model, the quantity $\Omega = \dot{\phi }+\dot{\psi }$, which is referred to as *gyricity*, can be approximated as $\Omega \approx \dot{\psi }$ for a rapidly rotating spinner. The positive direction of spin is chosen as in Fig. 5, i.e. anticlockwise relative to the $z'$-axis, where $(x',y',z')$ are the local coordinates (see Fig. 5a).

The equations of motion for the transverse displacement components at $z=L$ have the form

22 $$\begin{aligned} \begin{pmatrix} \ddot{U}(t)\\ \ddot{V}(t) \end{pmatrix}+ \frac{I_1}{I_0}\Omega {\textbf {R}}\begin{pmatrix} \dot{U}(t)\\ \dot{V}(t) \end{pmatrix}+ \frac{mgL}{I_0}\begin{pmatrix} U(t)\\ V(t) \end{pmatrix}= \begin{pmatrix} 0 \\ 0 \end{pmatrix}, \end{aligned}$$

where the $90^\circ$ rotation matrix ${\textbf {R}}$, which couples *U*(*t*) and *V*(*t*), is given by (4). Here, *g* is the acceleration due to gravity, $I_0$ is the transverse moment of inertia, relative to the pivot point at $z=0$, of the rigid rod combined with the spinner with respect to the $x'$-axis, which is assumed to be the same as for the $y'$-axis; the quantity $I_1$ is the moment of inertia of the spinner relative to its $z'$-axis.

Figure 5b shows an illustrative comparison between experimental measurements and the analytical solution, based on the linearised model. The movie of the motion of the gyropendulum is taken from underneath the gyropendulum, with the initial velocities being zero. The trajectory is shown in white, and, as discussed in Full classification for the transient motion of the gravitational spinner section, this case corresponds to Class 2 of transient motions with cusps. The parameter values used in the experimental setup are: the length of the pendulum arm is 0.58 m, and the radius, gyricity and mass of the disc are 0.06 m, $-172$ rad/s and $m=0.032$ kg, respectively. In addition, the initial displacement prescribed along the *x*-direction is $-0.12857$ m, while the initial displacement in the *y*-direction is set to $-0.00602$ m. As seen from Fig. 5b, the agreement with the analytical results is very good.

### Normalisation and chirality of the system

Assuming that the length of the arm of the pendulum is much larger than the radius of the spinner and that the spinner is represented by a uniform thin disc, the system (22) becomes

23 $$\begin{aligned} \begin{pmatrix} \ddot{U}(t)\\ \ddot{V}(t) \end{pmatrix}+ \frac{\Omega R^2}{2 L^2} {\textbf {R}}\begin{pmatrix} \dot{U}(t)\\ \dot{V}(t) \end{pmatrix}+ \frac{g}{L}\begin{pmatrix} U(t)\\ V(t) \end{pmatrix}= \begin{pmatrix} 0 \\ 0 \end{pmatrix}. \end{aligned}$$

It is noted in passing that Eq. (23) are analogous to those which describe the vibrations of an electron in a magnetic field as pointed out in^7,9^, in the context of the gyrostatic analogue of the Lorentz force (see^2^). It is convenient to introduce the dimensionless time $\tilde{t}=t\sqrt{g/L}$ to discuss the general motion of the gyropendulum for a variety of different initial conditions at the end of the rod ($z=L$). Equation (23) then become

24 $$\begin{aligned} \frac{d^2}{d\tilde{t}^2}\begin{pmatrix} \tilde{U}\\ \tilde{V} \end{pmatrix}+ \Gamma \begin{pmatrix} 0 &{} 1\\ -1 &{} 0 \end{pmatrix} \frac{d}{d\tilde{t}}\begin{pmatrix} \tilde{U}\\ \tilde{V} \end{pmatrix}+ \begin{pmatrix} \tilde{U}\\ \tilde{V} \end{pmatrix}= \begin{pmatrix} 0 \\ 0 \end{pmatrix}, \end{aligned}$$

where $$\Gamma =\Omega R^2/2\sqrt{gL^3}$$, $$\tilde{U}(\tilde{t})=U(\tilde{t}\sqrt{L/g})$$ and $$\tilde{V}(\tilde{t})=V(\tilde{t}\sqrt{L/g}).$$

Assuming solutions of the form $\tilde{U}=A\exp (i \tilde{\omega } \tilde{t})$, $\tilde{V}=B\exp (i \tilde{\omega } \tilde{t})$, and substituting into the system (24), then the solvability condition to find non-trivial solutions of the system gives four discrete dimensionless frequencies as

25 $$\begin{aligned} \begin{gathered} \tilde{\omega }_{1}=-\frac{1}{2}\Big (\Gamma +\sqrt{\Gamma ^2+4}\Big ), ~ \tilde{\omega }_{2}=\frac{1}{2}\Big (\Gamma -\sqrt{\Gamma ^2+4}\Big ), \\ \tilde{\omega }_{3}=\frac{1}{2}\Big (-\Gamma +\sqrt{\Gamma ^2+4}\Big ),~ \tilde{\omega }_{4}=\frac{1}{2}\Big (\Gamma +\sqrt{\Gamma ^2+4}\Big ). \end{gathered} \end{aligned}$$

For any real value of $\Gamma$, the quantities $\tilde{\omega }_{1}$ and $\tilde{\omega }_{2}$ are negative while $\tilde{\omega }_{3}$ and $\tilde{\omega }_{4}$ are positive. By choosing the positive eigenfrequencies $\tilde{\omega }_3$ and $\tilde{\omega }_4$, we write the corresponding eigenvectors $\textbf{u}_{3}=(1, i)^T$ and $\textbf{u}_{4}=(1, - i)^T$, respectively. Here, the general solution of (24) is a linear combination of four complex exponential functions; each depending on one of the four eigenvalues (25), with two of the eigenvalues being positive and two negative. In view of (25), the solution has been reduced to a form dependent on only two of these eigenvalues. The choice made in the text below (see (27)), is to use the two positive eigenvalues since these physically may be interpreted as real positive frequencies.

For the two positive values $\tilde{\omega }_{3}$ and $\tilde{\omega }_{4},$ it may be seen from (25) that in the absence of gyricity (i.e. when $\Gamma =0$), $\tilde{\omega }_{3}=\tilde{\omega }_{4}=1.$ Hence, in this particular case the motion becomes time-harmonic with the unit radian frequency, and the corresponding trajectory of the motion has an elliptical shape. Conversely, with the introduction of non-zero gyricity (i.e. when $\Gamma \ne 0$), the values of $\tilde{\omega }_{3}$ and $\tilde{\omega }_{4}$ become different. Furthermore, the difference between $\tilde{\omega }_{3}$ and $\tilde{\omega }_{4}$ grows with increasing gyricity, i.e. $|\tilde{\omega }_{4} - \tilde{\omega }_{3}| = |\Gamma |.$

**Figure 6:**
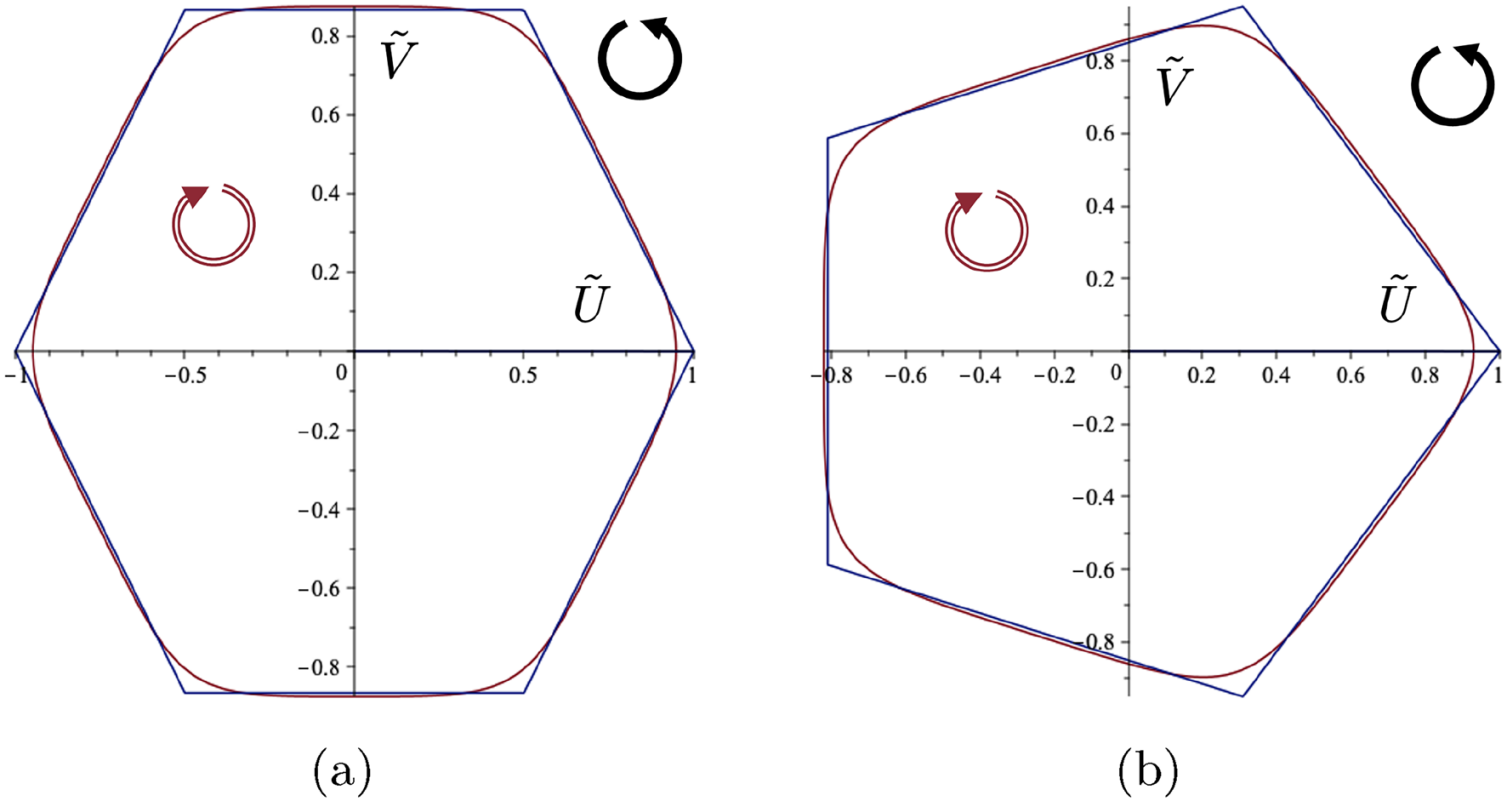
The approximations to the prescribed hexagonal and pentagonal orbits. The calculated values of $\Gamma$ and the initial conditions to generate these orbits are (**a**) $\Gamma =1.78885, U_0=0.9484$, $V_0=0, \dot{U_0}=0, \dot{V_0}= -0.326248$, and (**b**) $\Gamma =3/2, U_0=0.9298, V_0=0$, $\dot{U_0}=0, \dot{V_0}= -0.3282$. The solid arrows show the direction of spin of the gyroscopic spinner, and the hollow arrows show the orientation of motion for each respective polygonal orbit of the gyropendulum.

The general solution of (24) may be found as a linear combination of the normal mode solutions. Applying the initial conditions

26 $$\begin{aligned} \tilde{U}(0)=\tilde{U}_{0}, \quad \tilde{V}(0)=\tilde{V}_0, \quad \frac{d {\tilde{U}}}{d \tilde{t}}(0)=\dot{\tilde{U}}_{0},\quad \frac{d {\tilde{V}}}{d \tilde{t}}(0)=\dot{\tilde{V}}_{0}, \end{aligned}$$

where $\tilde{U}_0,\tilde{V}_0,\dot{\tilde{U}}_{0}$ and $\dot{\tilde{V}}_{0}$ are given values of the normalised initial displacements and initial velocities, then the solution of (24) with the initial conditions (26) becomes

27 $$\begin{aligned} \begin{pmatrix} \tilde{U} \\ \tilde{V} \end{pmatrix} = \tilde{A}_1\begin{pmatrix} \cos (\tilde{\omega }_4 \tilde{t}) \\ \sin (\tilde{\omega }_4 \tilde{t}) \end{pmatrix}+ \tilde{A}_2\begin{pmatrix} -\sin (\tilde{\omega }_4 \tilde{t}) \\ \cos (\tilde{\omega }_4 \tilde{t}) \end{pmatrix} + \tilde{A}_3\begin{pmatrix} \cos (\tilde{\omega }_3 \tilde{t}) \\ -\sin (\tilde{\omega }_3 \tilde{t}) \end{pmatrix}+ \tilde{A}_4\begin{pmatrix} \sin (\tilde{\omega }_3 \tilde{t}) \\ \cos (\tilde{\omega }_3 \tilde{t}) \end{pmatrix}, \end{aligned}$$

where

28 $$\begin{aligned} \tilde{A}_1 = \frac{\dot{\tilde{V}}_{0}+\tilde{\omega }_3 \tilde{U}_{0}}{\tilde{\omega }_3+\tilde{\omega }_4}, \quad \tilde{A}_2 = \frac{-\dot{\tilde{U}}_{0}+\tilde{\omega }_3 \tilde{V}_0}{\tilde{\omega }_3+\tilde{\omega }_4}, ~~~\tilde{A}_3 = \frac{-\dot{\tilde{V}}_{0}+\tilde{\omega }_4 \tilde{U}_{0}}{\tilde{\omega }_3+\tilde{\omega }_4}, \quad \tilde{A}_4 = \frac{\dot{\tilde{U}}_{0}+\tilde{\omega }_4 \tilde{V}_{0}}{\tilde{\omega }_3+\tilde{\omega }_4}. \end{aligned}$$

Thus each component of the displacement consists of a linear combination of two sinusoidal motions. It may also be seen from (25) to (28) that the initial conditions do not influence the frequency components, but they define the coefficients $\tilde{A}_{j},$ for $j=1,2,3,4$ (see (28)). The chirality parameter $\Gamma$ affects the frequency components and hence the coefficients $\tilde{A}_{1},\tilde{A}_{2},\tilde{A}_{3}$ and $\tilde{A}_{4}.$

### “Paradox” of pentagonal and hexagonal shapes

The longstanding Voyager programme, together with the Cassini programme, have delivered interesting observations of the hexagonal patterns on the North Pole of Saturn. In 2012, the NASA Cassini probe captured a fascinating movie of a storm at the North Pole of Saturn. Despite an expectation of circular profiles of vortex motion, the shape captured by the probe was hexagonal^5^. The analysis of these observations was published in^25,26^. Furthermore, Rossby wave patterns in the higher levels of the Earth’s atmosphere resembled a perturbed pentagon^4^. Both pentagonal and hexagonal patterns were observed at the South Pole of Jupiter^6^. An experimental demonstration^27,28^, of liquid vortex sloshing in a rotating cylindrical container, has also confirmed the presence of polygonal patterns, including pentagonal and hexagonal shapes. Although looking like a paradox, the question of formation of polygonal shapes can be answered. Without going into complexity of rotational fluid flows, we can simply note that in addition to a rotational action, there is also an additional force, which is gravity.

The elementary gravitational spinner (or a gyropendulum), in the linearised settings, takes into account both the rotational action as well as the action of gravity. Can a gyropendulum be designed so that it traces a prescribed periodic trajectory possessing a degree of rotational symmetry? The answer is in the affirmative.

In order to cause the gyropendulum to move in a prescribed trajectory, the parameter $\Gamma$ needs to be determined together with the knowledge of how to set the gyropendulum in motion, i.e. the initial conditions. For polygonal shapes, this may be done to a very good degree of approximation. The solution (27) may be regarded as a Fourier series with two frequency components and without the frequency-independent term. It is convenient to write this in complex notation as

29 $$\begin{aligned} z(\tilde{t})=c_1 e^{-i\tilde{\omega }_3\tilde{t}}+c_2 e^{i\tilde{\omega }_4\tilde{t}}, \end{aligned}$$

and require that the ratio $\tilde{\omega }_4/\tilde{\omega }_3$ is an integer related to the degree of rotational symmetry of the polygon. This requirement fixes the value of the parameter $\Gamma$. Suitable initial conditions may be found such that the complex constants $c_1$ and $c_2$ in (29) correspond to the Fourier coefficients for the particular polygon, as illustrated in Fig. 6.

Two examples are shown in Fig. 6 where a prescribed (a) hexagon and (b) pentagon may be seen together with their approximating trajectories.

The Fourier coefficients in these two cases are (a) $c_1=0.9119$ and $c_2=0.0365$, and (b) $c_1=0.8751$ and $c_2=0.0547$. The consequent values of the parameter $\Gamma$ together with the initial conditions are shown in the figure caption. There is very good agreement between the two prescribed shapes and their approximating trajectories considering a two component truncated Fourier series only is available from the analytical solution. It is also noted that the ratio $c_2/c_1$ is small in both cases illustrating how these polygonal shapes are a perturbation of the basic circular motion.

**Figure 7:**
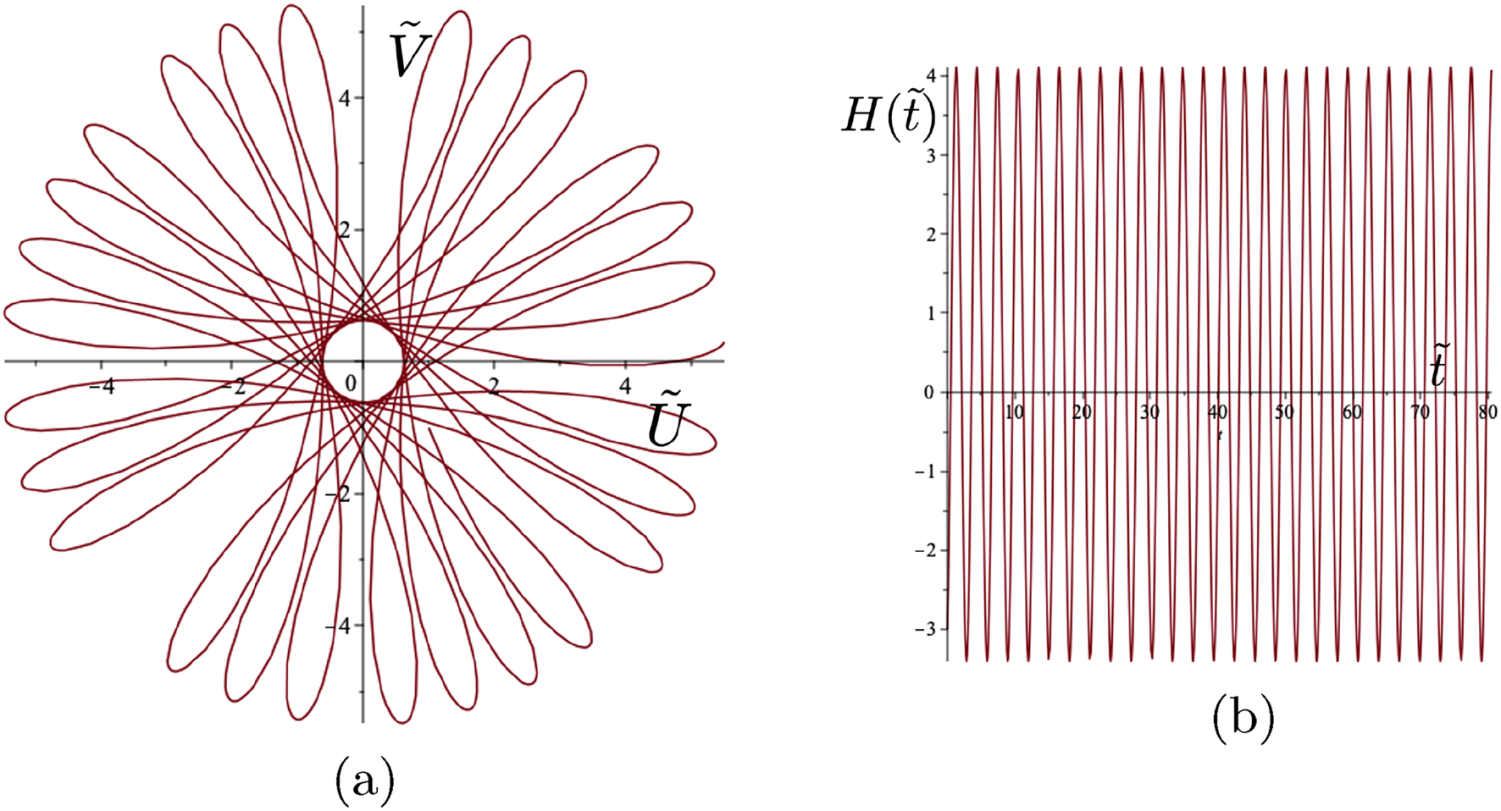
Class 1 example. Gyropendulum trajectory with $\Gamma =0.5$ and initial conditions $\tilde{U}_0 = 1$, $\tilde{V}_0 = -1$, $\dot{\tilde{U}}_0 = 2$, $\dot{\tilde{V}}_0 = -5$. (**a**) The self-intersecting trajectories around the origin of variable orientation. (**b**) The corresponding function $H(\tilde{t})$. Here the calculated class values are $M=-7.25$, $N=0.5$ and $H(0)=-3$ in accordance with (36).

### Full classification for the transient motion of the gravitational spinner

The trajectories of the gyropendulum may have different shapes, including smooth curves, self-intersecting loops, cusps, and they are dependent on the initial conditions and the given value of $\Gamma$.

**Figure 8:**
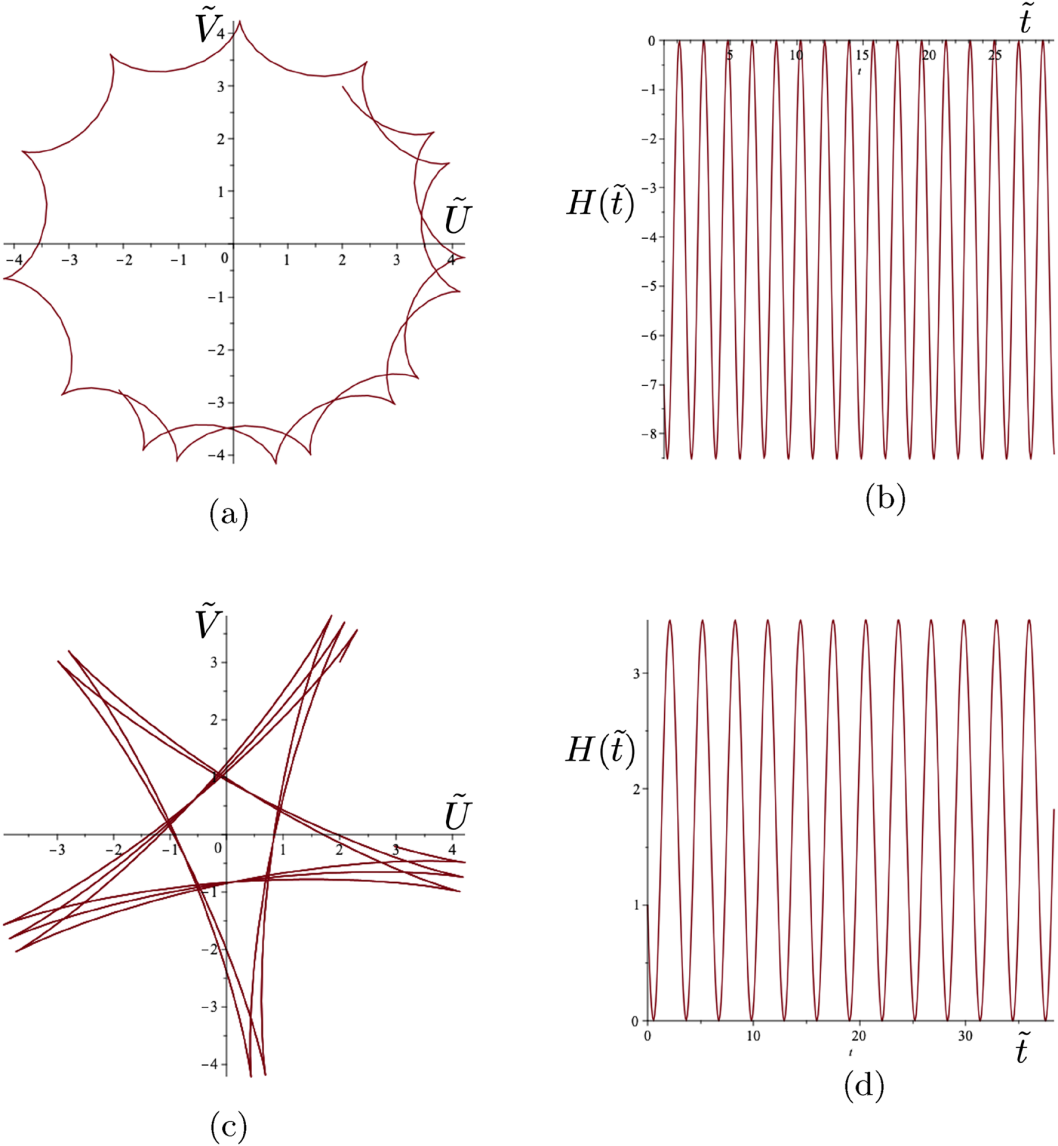
Class 2(a) examples. (**a**) Cusp trajectory of the gyropendulum and (**b**) the function $H(\tilde{t})$ with $\Gamma =2.8$ and initial conditions $\tilde{U}_0 =2$, $\tilde{V}_0 = 3$, $\dot{\tilde{U}}_0 =1$, $\dot{\tilde{V}}_0 = -2$. The condition for cusps is satisfied with $H(0)=-\Gamma |\dot{\tilde{{\textbf {Q}}}}(0)|^2/2=-7$. Here $H(\tilde{t})\le 0.$ (**c**) Cusp trajectory of the gyropendulum and (**d**) the function $H(\tilde{t})$ with $\Gamma =-0.4$ and initial conditions $\tilde{U}_0 =2$, $\tilde{V}_0 = 3$, $\dot{\tilde{U}}_0 =1$, $\dot{\tilde{V}}_0 = 2$. The condition for cusps is satisfied with $H(0)=-\Gamma |\dot{\tilde{{\textbf {Q}}}}(0)|^2/2=1$. Here $H(\tilde{t})\ge 0$.

Based on the consideration of the angular momentum of a unit mass (with no gyricity) moving in the same plane as the pendulum at $z=L$, the scalar quantity $H(\tilde{t})$ is introduced to characterise the orientation of the motion of the gyropendulum:

30 $$\begin{aligned} H(\tilde{t}){\textbf {k}}= (\tilde{U}(\tilde{t})\dot{\tilde{V}}(\tilde{t})-\tilde{V}(\tilde{t})\dot{\tilde{U}}(\tilde{t})) {\textbf {k}}= \tilde{{\textbf {Q}}}(\tilde{t})\times \dot{\tilde{{\textbf {Q}}}}(\tilde{t}), \end{aligned}$$

where ${\textbf {k}}$ is the basis vector along the *z*-axis, and $\tilde{{\textbf {Q}}}(\tilde{t})=(\tilde{U}(\tilde{t}),\tilde{V}(\tilde{t})).$ The scalar quantity $H(\tilde{t})$ is related to the magnitude of the angular momentum of the system. Whilst the position and velocity at any time in the motion are clearly seen on the trajectory, the quantity $H(\tilde{t})$ is a characterising single measure, reliant on both the position and velocity at any time.

Substituting the solution (27) and (28) into (30), leads to the following sinusoidal form for the function $H(\tilde{t})$

31 $$\begin{aligned} H(\tilde{t})=a+b \cos (\Xi \tilde{t} -\chi ), \end{aligned}$$

where

32 $$\begin{aligned} \begin{gathered} a=\frac{[|\dot{\tilde{{\textbf {Q}}}}(0)|^2-|\tilde{{\textbf {Q}}}(0)|^2]\Gamma +4H(0)}{\Xi ^2}, ~~~~b=\frac{\sqrt{P^2+R^2}}{\Xi ^2}, \\ \Xi =\sqrt{\Gamma ^2+4}, ~~~~P=\Gamma (\Xi \tilde{{\textbf {Q}}}(0) \cdot \dot{\tilde{{\textbf {Q}}}}(0)),~~~~~R=\Gamma (H(0)\Gamma +|\tilde{{\textbf {Q}}}(0)|^2-|\dot{\tilde{{\textbf {Q}}}}(0)|^2), \\ \chi =\tan ^{-1}\Bigg ({ \frac{P}{R}\Bigg ) ~ \text{ for }~ ~~R > 0, ~~~\chi =\pi +\tan ^{-1}\Bigg (\frac{P}{R}\Bigg ) ~ \text{ for } ~~ R < 0, ~~\chi = {\text {sgn}}(P) \frac{\pi }{2} ~\text{ for } ~ R=0.} \end{gathered} \end{aligned}$$

The sign of $H(\tilde{t})$ determines the orientation of the gyropendulum trajectory about the origin.

It is noted that when $\Gamma =0$, the function $H(\tilde{t})$ becomes constant and $H(\tilde{t}){\textbf {k}}=H(0){\textbf {k}}=\tilde{{\textbf {Q}}}(0)\times \dot{\tilde{{\textbf {Q}}}}(0)$, indicating the conservation of angular momentum for a particle of unit mass moving in the plane $z=L$ as expected.

The question arises as to whether the quantity $H(\tilde{t})$ may be constant for any non-zero value of $\Gamma$. This happens for the special case of degeneracy, when $P=R=0$, the quantity *H* is independent of $\tilde{t}$, i.e. $H=a$, as follows. When $\Gamma \ne 0$, one can identify the initial displacement and velocity, for which *H* remains constant for any value of $\tilde{t}.$ In this case, it follows from (30) and (32) that for non-zero *H*, and $P=0$, the vectors $\tilde{{\textbf {Q}}}(0)$ and $\dot{\tilde{{\textbf {Q}}}}(0)$ are orthogonal, and $|H(0)| = |\tilde{{\textbf {Q}}}(0)| |\dot{\tilde{{\textbf {Q}}}}(0)|$. Furthermore, it follows from (32) and $R=0$ that $|\tilde{{\textbf {Q}}}(0)| |\dot{\tilde{{\textbf {Q}}}}(0)| \Gamma {\text {sgn}}(H(0)) +|\tilde{{\textbf {Q}}}(0)|^2 - |\dot{\tilde{{\textbf {Q}}}}(0)|^2 = 0$, which leads to $|\tilde{{\textbf {Q}}}(0)| /|\dot{\tilde{{\textbf {Q}}}}(0)|=\frac{1}{2} (\omega - \Gamma {\text {sgn}}(H(0))).$ The corresponding trajectories of the gyropendulum have circular shapes (of different orientations depending on ${\text {sgn}}(H(0))$), and the quantity *H* is constant.

The sign of the product of the maximum and minimum values of $H(\tilde{t})$ is used to determine the classes of trajectories for given values of $\Gamma , \tilde{{\textbf {Q}}}(0)$ and $\dot{\tilde{{\textbf {Q}}}}(0).$ It may be shown that this product is given by

33 $$\begin{aligned} a^2-b^2 = -\frac{\Big (\Gamma |\tilde{{\textbf {Q}}}(0)|^2-2H(0)\Big )\Big (\Gamma |\dot{\tilde{{\textbf {Q}}}}(0)|^2+2H(0)\Big )}{\Gamma ^2+4}. \end{aligned}$$

Hence the classification of the forms of the trajectories is linked to the initial value *H*(0) and to the interval $\Upsilon =(M,N)$ where

34 $$\begin{aligned} M = -\Gamma |\dot{\tilde{{\textbf {Q}}}}(0)|^2/2, ~~N= \Gamma |\tilde{{\textbf {Q}}}(0)|^2/2,&~~ \Gamma >0, \end{aligned}$$

35 $$\begin{aligned} M= \Gamma |\tilde{{\textbf {Q}}}(0)|^2/2, ~~N= -\Gamma |\dot{\tilde{{\textbf {Q}}}}(0)|^2/2,&~~\Gamma < 0. \end{aligned}$$

**Figure 9:**
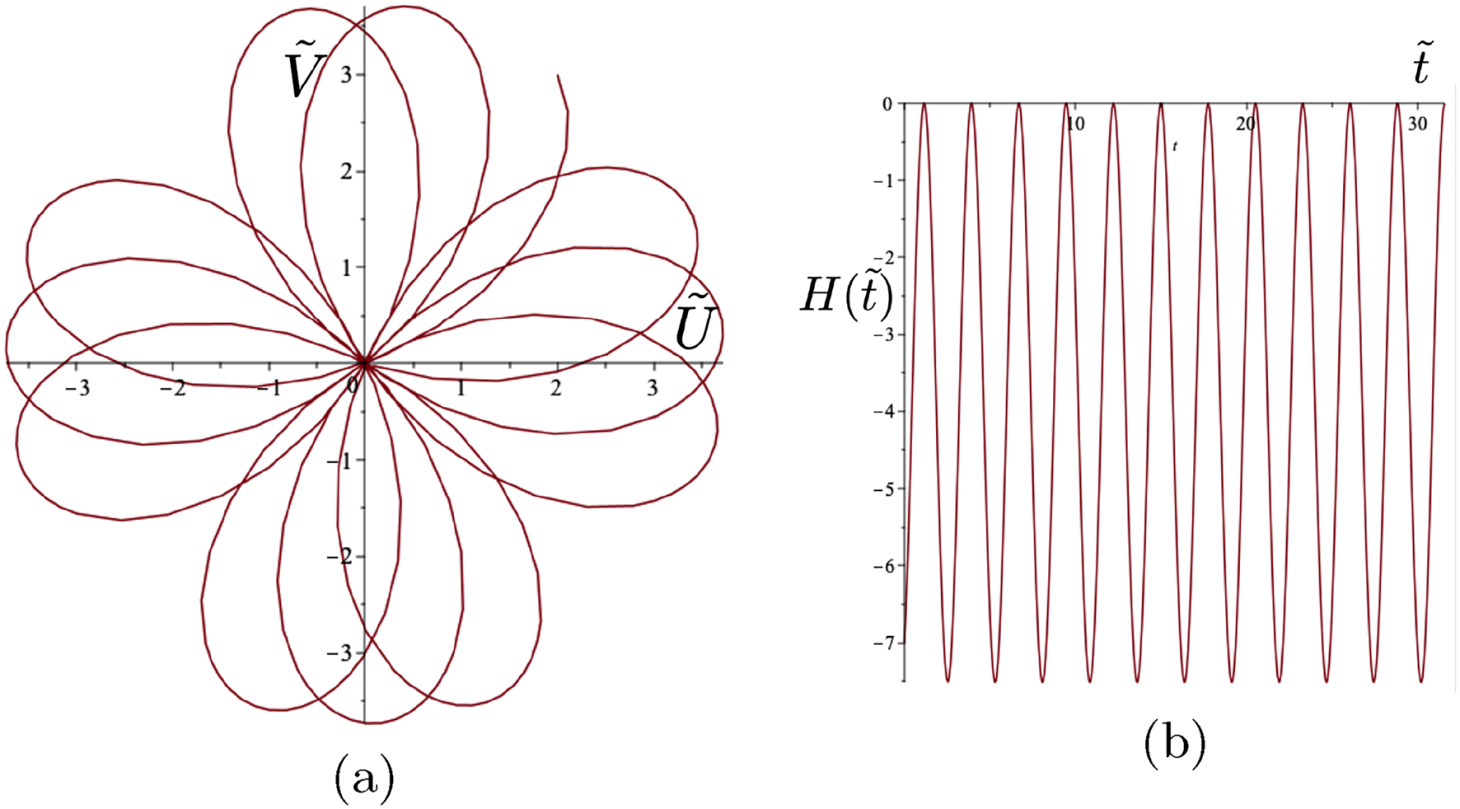
Class 2(b) example. (**a**) Trajectory of the gyropendulum with smooth loops passing through the origin and (**b**) the function $H(\tilde{t})$ with $\Gamma =-1.077$ and initial conditions $\tilde{U}_0 =2$, $\tilde{V}_0 = 3$, $\dot{\tilde{U}}_0 =1$, $\dot{\tilde{V}}_0 = -2$. The condition for Class 2(b) is satisfied with $H(0)=\Gamma |\tilde{{\textbf {Q}}}(0)|^2/2=-7$. In addition $H(\tilde{t}) \le 0$.

The characterising function $H(\tilde{t})$ (see (30)), whilst always sinusoidal, may be always positive, always negative or zero at its maximum or minimum. This function is strongly influenced by the value of the gyricity through the solution for the displacement (25)–(28) and the corresponding velocity. Such differences in behaviour of this characterising function leads to different types of motion. *Three classes of trajectories* can be identified as follows. **Class 1** incorporates the cases when $H(0) \in \Upsilon$, **Class 2** corresponds to $H(0) \in \partial \Upsilon$, and **Class 3** includes all configurations where $H(0) \notin \overline{\Upsilon }$. It should be noted that *H*(0), *M* and *N*,  which feature in the classification constraints, indicate the allowable combinations of the fundamental parameters $\Gamma$, $\tilde{U}_0$, $\tilde{V}_0$, $\dot{\tilde{U}}_0$ and $\dot{\tilde{V}}_0$ for the various classes.

**Figure 10:**
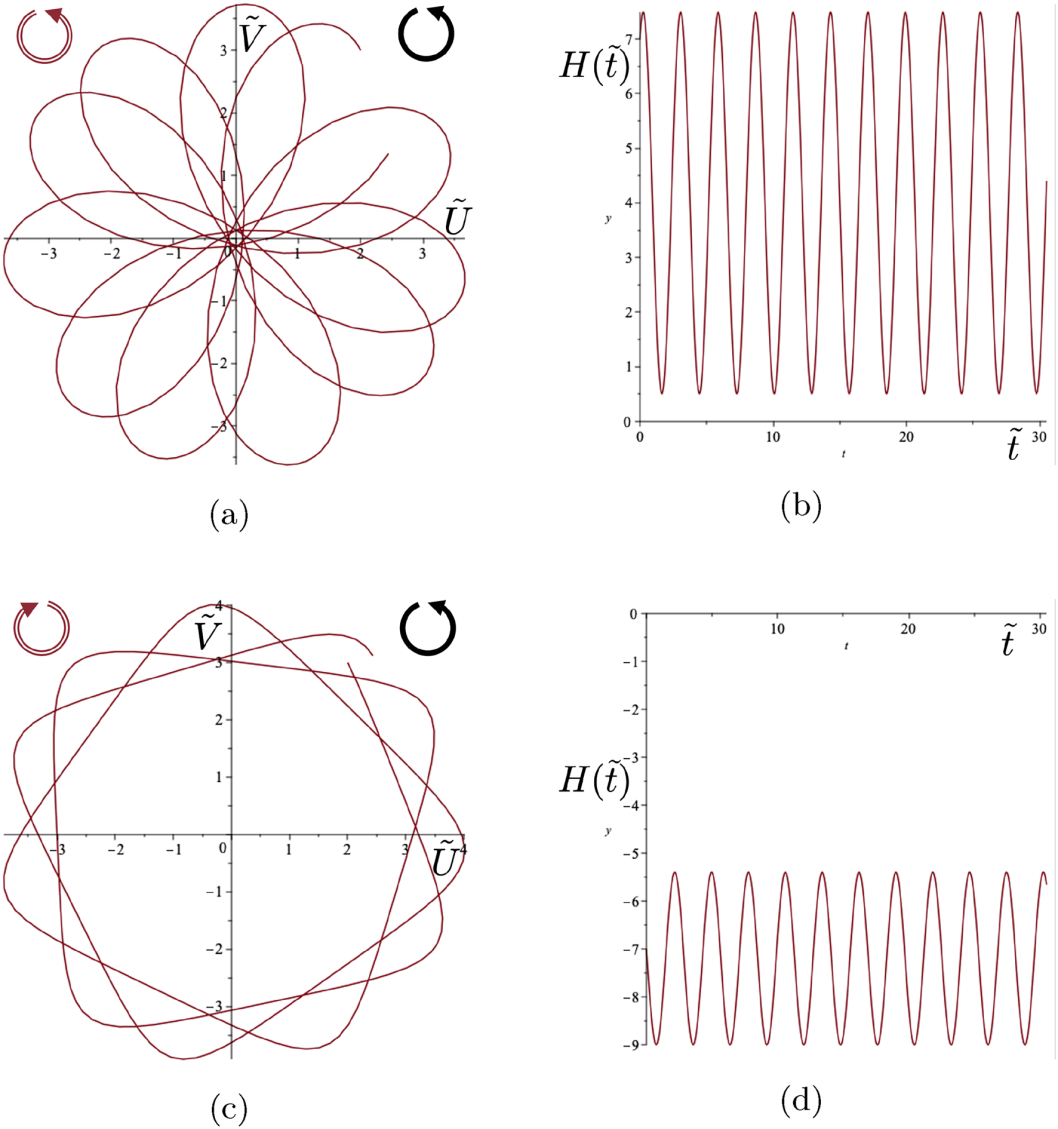
Class 3 examples. (**a**) Smooth trajectories of the gyropendulum not passing through the origin, and (**b**) the function $H(\tilde{t})$ with parameter values $\Gamma =1$ and initial conditions $\tilde{U}_0 =2$, $\tilde{V}_0 = 3$, $\dot{\tilde{U}}_0 =-1$, $\dot{\tilde{V}}_0 = 2$. The Class 3 conditions are satisfied with $H(0)=7$, $- \Gamma |\dot{\tilde{{\textbf {Q}}}}(0)|^2/2=-2.5$ and $\Gamma |{\tilde{{\textbf {Q}}}}(0)|^2/2=6.5.$ (**c**) Smooth trajectories of the gyropendulum not passing through the origin, and (**d**) the function $H(\tilde{t})$ with parameter values $\Gamma =1$ and initial conditions $\tilde{U}_0 =2$, $\tilde{V}_0 = 3$, $\dot{\tilde{U}}_0 =1$, $\dot{\tilde{V}}_0 = -2$. The Class 3 conditions are satisfied with $H(0)=-7$, $- \Gamma |\dot{\tilde{{\textbf {Q}}}}(0)|^2/2=-2.5$ and $\Gamma |{\tilde{{\textbf {Q}}}}(0)|^2/2=6.5.$ The solid arrows indicate the direction of spin of the gyroscopic spinner, and the hollow arrows show the orientation of motion for each respective trajectory of the gyropendulum.

- *Class 1*: *self-intersecting loops of variable orientation* occur when
  36 $$\begin{aligned} M<H(0)<N . \end{aligned}$$
  The motion of the gyropendulum corresponds to the alternating orientation about the origin during the motion. An example of such a trajectory with self-intersecting loops and the corresponding graph of $H(\tilde{t})$ are shown in Fig. 7. The orientation of the trajectory of the gyropendulum will alternate between clockwise and anticlockwise motion relative to the origin according to the sign changes of the function $H(\tilde{t})$.
- *Class 2*: For this class, the quantity (33) equals zero. There are two sub-classes:
  **(a)** Trajectories have *cusps* when the motion does not change its orientation (i.e. $H(\tilde{t})$ does not change sign), and the following condition is satisfied
  37 $$\begin{aligned} H(0)=-\Gamma |\dot{\tilde{{\textbf {Q}}}}(0)|^2/2. \end{aligned}$$
  The trajectories of the gyropendulum, which contain cusps, do not pass through the origin. The function $H(\tilde{t})$ equals zero only at the points corresponding to the cusps, where the velocity vanishes. Two typical trajectories involving cusps, together with the corresponding graphs of $H(\tilde{t})$, are shown in Fig. 8a–d, with clockwise and anticlockwise trajectories around the origin, respectively.
  **(b)** *Loops passing through the origin* occur when the motion does not change its orientation (i.e. $H(\tilde{t})$ does not change sign), and the following condition is satisfied
  38 $$\begin{aligned} H(0)=\Gamma |\tilde{{\textbf {Q}}}(0)|^2/2. \end{aligned}$$
  The trajectories have no cusps present, and the function $H(\tilde{t})$ vanishes only at the points corresponding to the origin. A typical trajectory is shown in Fig. 9a with the corresponding function $H(\tilde{t})$ shown in Fig. 9b.
- *Class 3*: Trajectories in this class are smooth curves, which do not pass through the origin, have no cusps ($H(\tilde{t}) \ne 0)$, and do not change their orientation. They are characterised by either $H(0)<M$ or $H(0)>N$ with clockwise or anticlockwise trajectories about the origin depending on the value of *H*(0) (see (34), (35)). Two related examples are shown in Fig. 10. Both have the same gyricity parameters and initial displacements, except for initial velocities in opposite directions resulting in opposite orientations about the origin. Class 3 also includes the case of polygonal trajectories, discussed in Sect. 3.2, with the examples of pentagonal and hexagonal shapes shown in Fig. 6.

## Concluding remarks

The study of gravitational spinners provides a formal connection between mechanical models and the models of electromagnetism and solid state physics, with similar phenomena being observed at different time scales and a different spatial scale. Chiral motion of planetary systems is similar to the motion of atomic structures within solids. Mathematical models, although limited on full generality of natural phenomena, can demonstrate formal connections between chiral features of gyroscopic forces combined with gravity at different scales.

### Supplementary Information

Supplementary Information 1.

Supplementary Information 2.

Supplementary Information 3.

Supplementary Information 4.

## Data Availability

All analytical data analysed during this study are included in this published article. The experimental data and video, related to Fig. 5b, are included in the electronic supplementary material provided for this article.
