# Supplementary material for: Controlling the motion of gravitational spinners and waves in chiral waveguides
Source: Sci Rep. 2024 Jan 12;14:1203. doi: 10.1038/s41598-023-50052-0 (PMC10786887; doi:10.1038/s41598-023-50052-0)
Supplement: Supplementary file 4 — Supplementary Information 4. [file 41598_2023_50052_MOESM4_ESM.docx]

**Supplementary material files**

There are three supplementary videos:

1. Supplementary Video V1

**Caption for Supplementary Video V1:** Animation of the gravity induced wave, shown in Fig. 1(a) of the paper.

1. Supplementary Video V2

**Caption for Supplementary Video V2:** Animation of the gravity induced wave in a gyroscopic waveguide, shown in Fig. 1(b) of the paper.

1. Supplementary Video V3

**Caption for Supplementary Video V3:** Video of the experiment, illustrated in Fig. 5(b) of the paper.
